# Drug response prediction using graph representation learning and Laplacian feature selection

**DOI:** 10.1186/s12859-022-05080-4

**Published:** 2022-12-09

**Authors:** Minzhu Xie, Xiaowen Lei, Jianchen Zhong, Jianxing Ouyang, Guijing Li

**Affiliations:** 1 College of Information Science and Engineering, Hunan Normal University, Changsha, China; 2 Key Laboratory of Computing and Stochastic Mathematics (LCSM) (Ministry of Education), School of Mathematics and Statistics, Hunan Normal University, Changsha, China

**Keywords:** Drug response, Learning graph representation, Laplacian feature selection, Network topology feature

## Abstract

**Background:**

Knowing the responses of a patient to drugs is essential to make personalized medicine practical. Since the current clinical drug response experiments are time-consuming and expensive, utilizing human genomic information and drug molecular characteristics to predict drug responses is of urgent importance. Although a variety of computational drug response prediction methods have been proposed, their effectiveness is still not satisfying.

**Results:**

In this study, we propose a method called LGRDRP (Learning Graph Representation for Drug Response Prediction) to predict cell line-drug responses. At first, LGRDRP constructs a heterogeneous network integrating multiple kinds of information: cell line miRNA expression profiles, drug chemical structure similarity, gene-gene interaction, cell line-gene interaction and known cell line-drug responses. Then, for each cell line, learning graph representation and Laplacian feature selection are combined to obtain network topology features related to the cell line. The learning graph representation method learns network topology structure features, and the Laplacian feature selection method further selects out some most important ones from them. Finally, LGRDRP trains an SVM model to predict drug responses based on the selected features of the known cell line-drug responses. Our five-fold cross-validation results show that LGRDRP is significantly superior to the art-of-the-state methods in the measures of the average area under the receiver operating characteristics curve, the average area under the precision-recall curve and the recall rate of top-*k* predicted sensitive cell lines.

**Conclusions:**

Our results demonstrated that the usage of multiple types of information about cell lines and drugs, the learning graph representation method, and the Laplacian feature selection is useful to the improvement of performance in predicting drug responses. We believe that such an approach would be easily extended to similar problems such as miRNA-disease relationship inference.

## Background

Personalized medicine focuses on finding appropriate drugs for individual patients. Since the same drugs have different effects on different patients, knowing the responses to drugs for each individual is a prerequisite of personalized medicine [1]. Since clinical drug response experiments are time-consuming and expensive, computational drug response prediction methods based on the related information of drugs and cell lines are of urgent practical importance and have attracted many researchers [2]. A variety of drug response prediction methods have been proposed, and they are mainly based on existing biological databases [3], of which Genomics of Drug Sensitivity in Cancer (GDSC) [4] and Cancer Cell Line Encyclopedia (CCLE) [5] are the two most famous. GDSC contains known cancer cell-drug responses and the corresponding cell lines’ profiles [4]. CCLE provides public access to the gene expression, gene methylation and mutation data of over 1100 cell lines [5]. These databases provide researchers with benchmark data to test drug response prediction methods.

Based on cell line gene expression data, Torkamani et al. [6] used PCA to extract gene expression features, and constructed a linear regression model to predict drug responses. Gupta et al. [7] proposed a prediction model based on genomic characteristics such as copy number variations of cancer cell lines. Based on the CCLE dataset, Fang et al. [8] used a quantile regression forest method to predict drug response. Based on a support vector machine and a recursive feature selection tool, Dong et al. [9] used the gene expression and drug sensitivity data in CCLE to build a drug response predictor. Using the same data set, Geeleher et al. [10] proposed a ridge regression prediction model. Liu et al. [11] proposed an ensemble learning method that integrated a low-rank matrix completion model and a ridge regression model to predict drug responses. By integrating the pathways of drug targets and the related gene sets, Ammad et al. [12] proposed a kernelized Bayesian matrix factorization with component-wise multiple kernel learning to predict drug responses.

Based on the gene expression features of cell lines and the chemical features of drugs, Li et al. [13] developed a deep learning architecture to learn a prediction model. Yan et al. [14] proposed an interpretable model to predict drug responses, which integrated drug features, cell line features and drug responses using triple matrix factorization, and Guvencpaltun et al. [15] proposed a framework of Bayesian importance-weighted tri-matrix and two-matrix factorization to predict drug responses. These methods mainly considered the basic information of cell lines and drugs, and obtained good prediction performance for some certain drugs. However, they neglected other useful information such as the relationship between different cell-lines and the relationship between different drugs [16].

Based on the assumption that similar cell-lines tend to respond similarly to similar drugs, a lot of network-based drug response prediction methods have been proposed recently. For example, after integrating different kinds of information such as

gene mutation, DNA copy number and mRNA expression data of cell lines and compound molecular properties, ATC-codes and side-effects of drugs, Wang et al. [17] built similarity networks for cell lines and drugs, and proposed an SVM classifier model. Stanfield et al. [18] integrated cell line gene mutations, known cell line-drug responses and protein-protein interactions (PPIs), and built a heterogeneous network consisting of the genes, cell lines and drugs. They utilized a random walk with restart (RWR) in the network to predict drug responses. Similarly, Zhang et al. [19] used cell line gene expression data to build a cell line similarity network, used drug chemical structures to build a drug similarity network, and used PPIs to build a gene-gene interaction network. They combined the networks with known cell line-drug associations and drug-target (gene) interactions into a heterozygous network and proposed a prediction model. Based on a cell line similarity network and a drug similarity network, Liu et al. [20] adopted a neighbor-based collaborative filtering with global effect removal method, Zhang et al. [21] adopted a hybrid interpolation weighted collaborative filtering method, and Guan et al. [22] utilized weighted graph regularized matrix factorization to predict drug responses.

In most network based drug response prediction methods, heterogeneous data are integrated using a weighted graph (i.e., a network), how to capture useful topological information of a graph is important for the efficiency of drug response prediction. Recently many learning graph representation methods have been introduced, and GraRep [23] is a state-of-the-art one that could learn a global representation of a graph, which contains the topological information of the graph and is convenient to use as input features of machine learning methods.

**Fig. 1:**
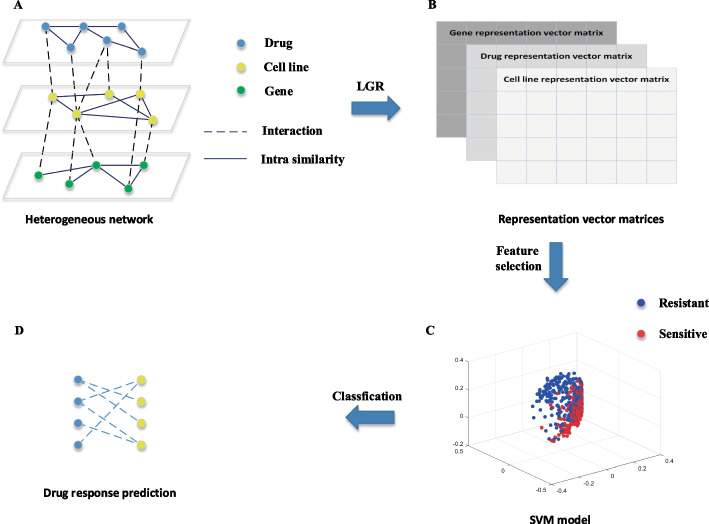
The flowchart of LGRDRP. LGRDRP consists of four steps:** A** Construction of a heterogeneous network.** B** Learning representation vectors using a learning graph representation (LGR) method.** C** Feature selection and SVM model training.** D** Drug response prediction

In the paper, we formulate the drug response prediction problem as a classification task as most of the existing methods: for each drug, classify cell lines into two groups: sensitive and resistant according to the cell lines’ features. To improve drug response prediction performance, we integrate several available related data such as known cell line-drug associations, miRNA expression profiles of cell lines, chemical structures of drugs, PPIs, cell line gene sequence variations and hypermethylation informtion into a heterogeneous network. Then GraRep [23] and Laplacian Feature Selection are used to learn the cell lines’ features in the network and features reduction, respectively. Finally, an SVM model [24] for the classification task is trained on the network. Our method is called LGRDRP (a Learning Graph Representation method for Drug Response Prediction) and is illustrated in Fig. 1.

## Results and discussion

We conducted a series of 5-fold cross-validation experiments to test the performance of LGRDRP and some other state-of-the-art drug response prediction methods. The cross-validations were done for each drug. When a query drug was selected, all the cell lines with known responses (sensitive or resistant) to the drug were randomly divided into 5 groups. We randomly select one group as the test data and the other four as the training data. The heterogeneous network of the train data was obtained by removing the edges between the query drug vertex and the test cell line vertices.

For each cell line, a drug response prediction method calculated a score, and the test cell lines were sorted according to their scores. With a fixed threshold, if the score of one cell line is below the threshold, it is labeled as negative (resistant), and if it is known sensitive to the query drug, it is a false negative; if it is known resistant to the drug, it is a true negative. When the prediction score of a cell line is equal to or above the threshold, it is viewed as positive, and if it is known sensitive to the query drug, it is a true positive; if it is known resistant to the drug, it is a false positive. The true positive rate (TPR), the false positive rate (FPR), the precision ratio (Prec) and the recall ratio (Rec) can be computed as follows: TPR = TP/(TP + FN), FPR = FP/(FP + TN), Prec = TP/(TP + FP), Rec = TP/(TP + FN), where TP, FN, FP and TN are the numbers of cell lines that are true positive, false negative, false positive and true negative, respectively.

With the threshold increases from the smallest score to the highest score, a receiver operating characteristic (ROC) curve is drawn according to the varying TPRs and FPRs as X-axis values and Y-axis values respectively.

The area under the ROC curve (AUC) is calculated to evaluate the prediction performance. Since in our data set, the number of resistant responses is much larger than the sensitive responses. To better measure the prediction performance, we also used another metric: the area under the precision-recall (PR) curve (AUPR). A PR curve is a trajectory of the performance at a plane with the precision ratio as Y-axis value and recall ratio as X-axis value when the threshold changes.

Furthermore we may be more interested in the cell lines at the top of the sorted list. Therefore, the percentages of the true sensitive cell lines in the top 10, 20, 50, 100 of the sorted cell lines according to their response scores to the query drug were also used to evaluate the prediction performance.

### Parameters selection

**Fig. 2:**
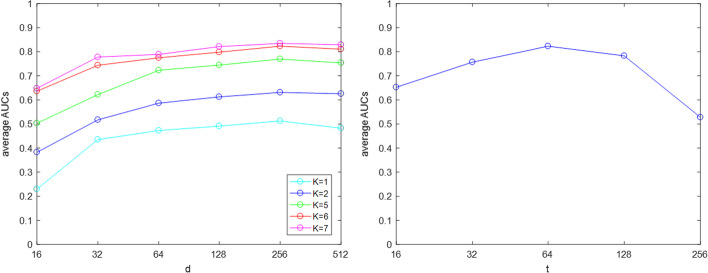
Performances of LGRDRP with different values of parameters *K*, *d* and *t*. The value of *t* is set 64 in the left panel, and $K = 6$ and $d = 256$ in the right panel

There are three parameters *K*, *d* and *t* need to set for LGRDRP, where *K* denotes the maximal transit steps, *d* determines the dimension of representation vectors and *t* is the number of features remained after the feature selection procedure. We tested the performance of LGRDRP with different *K*, *d* and *t*. Figure 2 shows the average AUC over different combinations of *K*, *d* and *t*. In the left panel, *t* was set 64. Please note, when the length $K \times d$ of the final representation vector is less than 64, all features are kept. It can be observed when *K* increased, the average AUC of LGRDRP increased accordingly. However, when *K* increased from 6 to 7, the performance of LGRDRP didn’t improve too much, but the computing time increased significantly. When *d* increased from 16 to 256, AUC increased accordingly. However, when *d* is set 512, AUC begins to decrease. The reason may be that the overfitting model results in poor generalization.

With $d = 256$ and $K = 6$, experiments were also conducted with varying *t*, and the results are shown in the right panel of Fig. 2. It indicates that the average AUC reached the best when $t = 64$. When *t* is too small, the left features can’t capture enough structure information, but when *t* is too large, too many features may include some unimportant information which may disturb the prediction ability [25].

In the following tests, $K = 6, d = 256$ and $t = 64$ without specific description.

### Performance evaluation

**Fig. 3:**
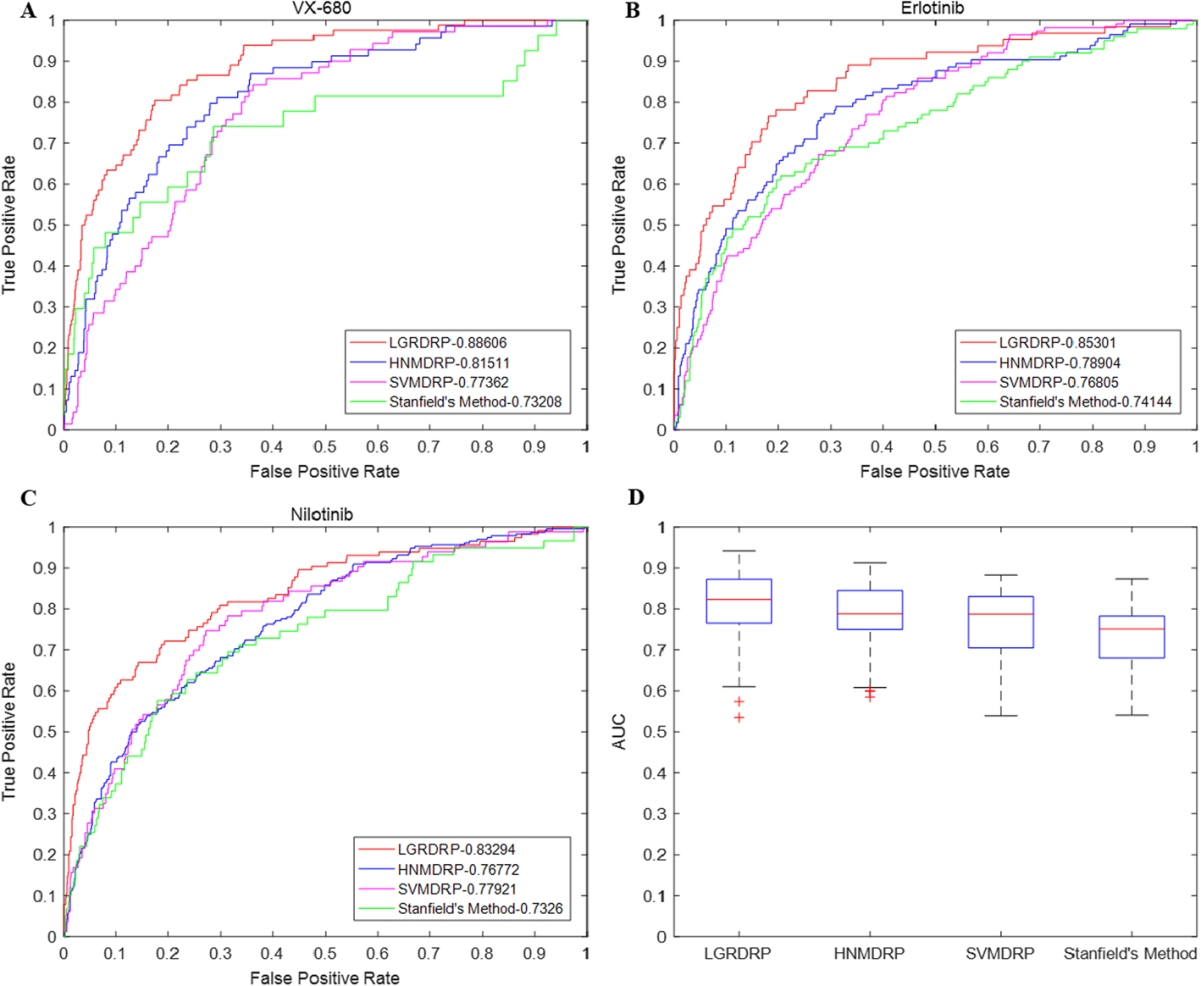
Prediction performances of LGRDRP and the other three methods (HNMDRP, SVMDRP and Stanfield’s method) based on their ROC curves and AUCs. **A**–**C** The ROC curves of four methods on drugs: VX-680, Erlotinib and Nilotinib, respectively.** D** The AUCs over all drugs

We compared the prediction performance of LGRDRP with three other art-of-the-state methods: HNMDRP [19], SVMDRP [17] and Stanfield’s method [18]. The parameters of HNMDRP, SVMDRP and Stanfield’s method were set as recommended by the corresponding literature. We compared their performances over 226 drugs of the GDSC dataset via the same five-fold cross-validation experiments, and the results are shown in Fig. 3. Figure 3A–C displays their ROC curves on three drugs: VX-680, Erlotinib and Nilotinib. It can be observed that for each case the ROC curve of LGRDRP is clearly above those of the others, which implies the prediction performance of LGRDRP is the best. Figure 3D illustrates the AUCs over all drugs with the comparison results reported as boxplots, which shows LGRDRP is generally more accurate than other methods. HNMDRP performs slightly better than SVMDRP, and SVMDRP better than Stanfield’s method, which indicates that protein information is not sufficient to reveal the cell line-drug association and integration of multi biological information could improve the predictive power. The average AUC of LGRDRP over all drugs is 0.8131, and achieves the highest value 0.9422 as regarding to drug SNX-2112, an oral anti-tumor drug as a Hsp90 inhibitor. For 50% drugs, the AUCs of LGRDRP are larger than 0.8229, and for 25% drugs, the AUCs of LGRDRP are larger than 0.8734.

**Fig. 4:**
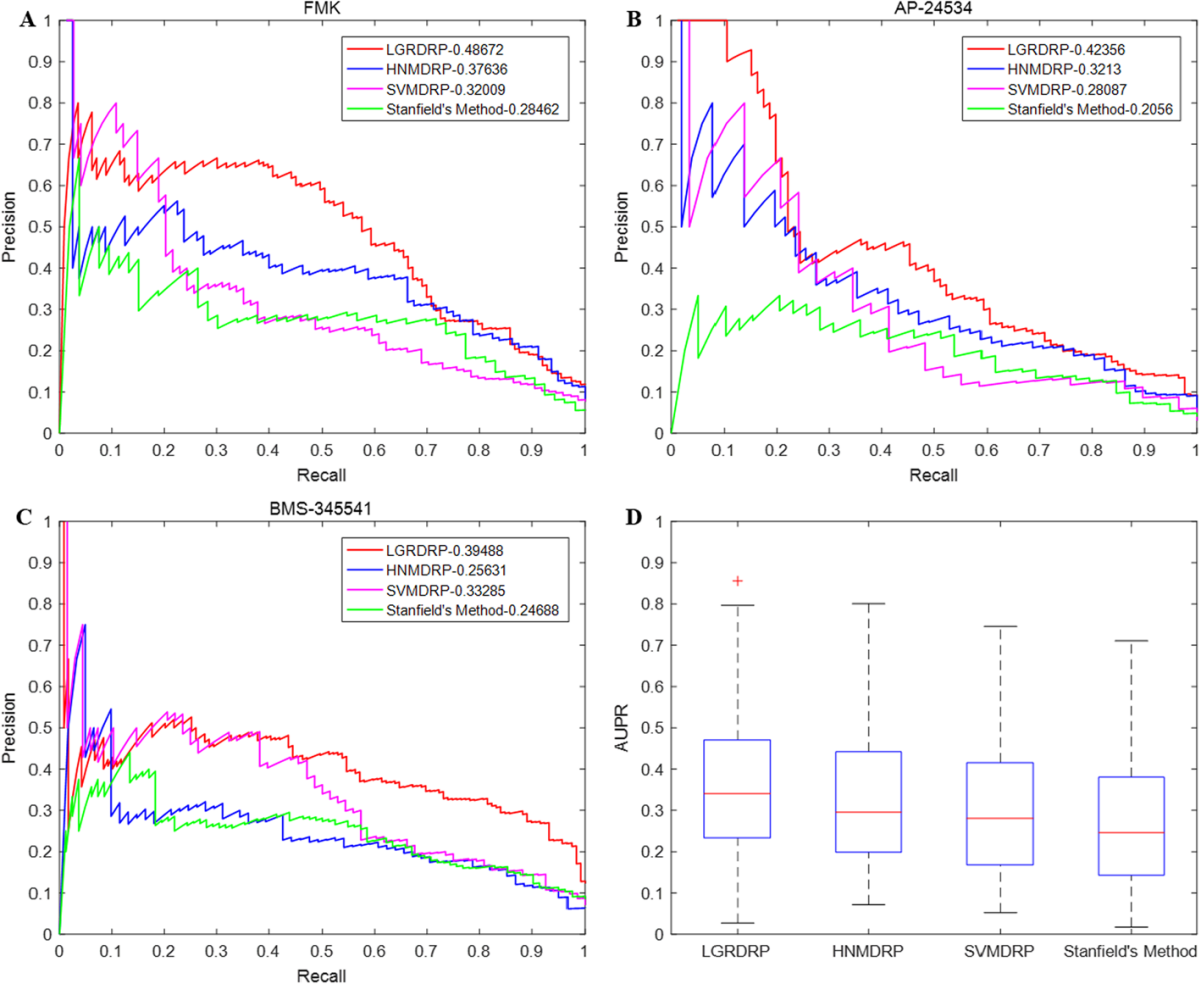
Prediction performances of LGRDRP, HNMDRP, SVMDRP and Stanfield’s method based on their PR curves and AUPRs.** A**–**C** The PR curves on drugs: FMK, AP-24534 and BMS-345541.** D** The AUPRs over all drugs

Figure 4A–C illustrates the PR curves of LGRDRP, HNMDRP, SVMDRP and Stanfield’s method on three drugs FMK, AP-24534 and BMS-345541. The PR curves of LGRDRP also lie above those of the other methods. The average AUPR of LGRDRP over all drugs is 8.52%, 11.63%, 16.79% higher than those of HNMDRP, SVMDRP and Stanfield’s method respectively. The AUPRs of the methods over all drugs are shown in Fig. 4D. The experiment results indicate that LGRDRP is successful to accomplish the prediction task even with the greatly unbalanced data set, demonstrating its reliability and prediction capability.

**Fig. 5:**
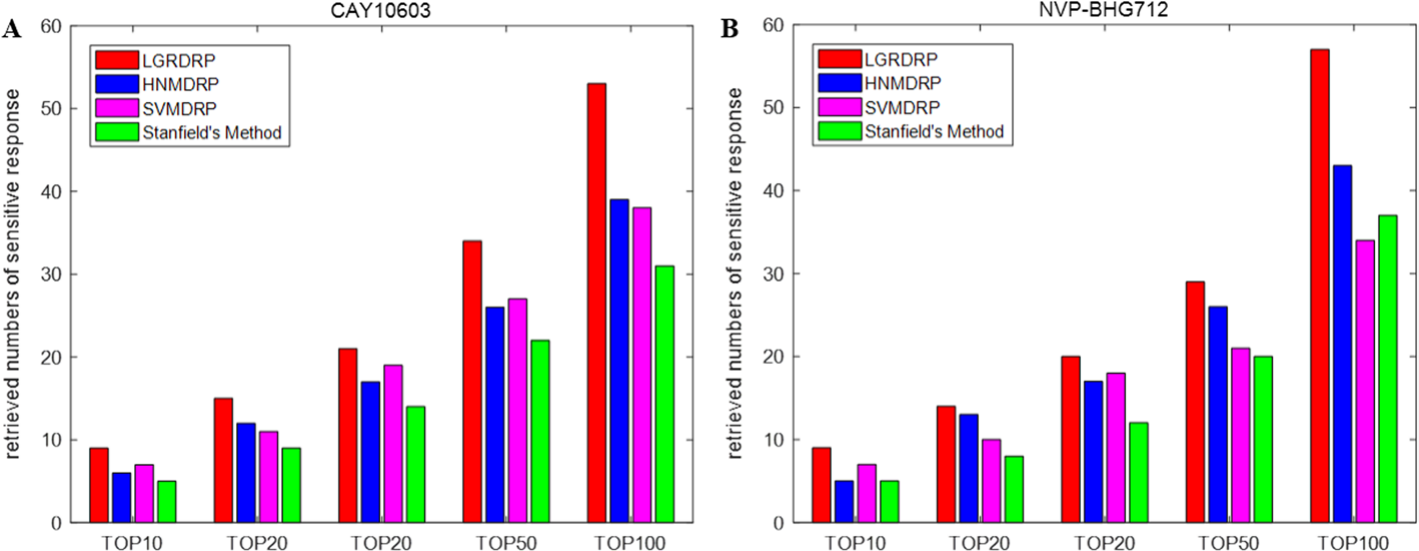
The retrieved number of sensitive response cell lines in the TOP10, TOP20, TOP30, TOP50, TOP100 predicted sensitive cell lines

Figure 5 shows the retrieved number of real sensitive cell lines in the predicted top 10, 20, 50, 100 sensitive cell lines for drugs CAY10603 and NVP-BHG712, and LGRDRPP shows a significant advantage over the other methods again.

### Conclusion

In this paper, we propose a drug responses prediction method called LGRDRP. It first uses the cell line miRNA expression profile to build a cell line similarity network, drug chemical structures to build drug similarity network, cell line gene variations and methylation data to build cell line-gene interaction network. By integrating the known cell line-drug responses into the above networks, LGRDRP constructs a heterogeneous network. Then LGRDRP uses a learning graph representation method GraRep to obtain the representation vectors as the topology structure features of vetices in the network. To avoid overfitting causing by using too many features, a Laplacian score method is adopted to pick out some important features. Finally, LGRDRP learns an SVM model which is used to predict drug responses. Extensive 5-fold cross-validation experiments showed that LGRDRP was generally superior to three art-of-the-state methods HNMDRP, SVMDRP and Stanfield’s method. The success of our method is based on the effective integration of diverse biological information, the good graph representation of the topology structure of the network, and the effective feature selection. After minor modifications or simple extensions, LGRDRP can also be employed in other biological predictions such as gene-disease [26], drug-target [27] and microRNA-disease [28], and the prediction performance can be further improved by admitting other appropriate biological information, such as gene function annotations and drug semantic annotations. In clinical practice, some combinations of multiple drugs can increase treatment efficacy, and the response prediction of a cell line response to a drug combination is an important extension of the single drug response prediction [29]. In the future, we are going to improve LGRDPR so that it could deal with the drug combination response prediction.

## Methods

### Construction of the heterogeneous network

The heterogeneous network consists of a drug similarity network, a cell line similarity network, a gene similarity network, a cell line-drug interaction network, and a cell line-gene interaction network, as shown in Fig. 1A.

The drug similarity network is based on the chemical structure data of 226 frequently used drugs, which consists of a 3D structure similarity matrix of the drugs and was downloaded from PubChem (http://pubchem.ncbi.nlm.nih.gov/). To avoid disturbing from noises and make sure the network has a clear biological meaning, the elements smaller than 0.2 in the similarity matrix were set as 0. The drug similarity network consists of 226 vertices and 24456 edges, where each vertex denotes a drug and each edge has a similarity score weight ($\ge 0.2$).

miRNA expression information of cell lines could be used to classify cancer cell lines into subtypes [30], and our cell line similarity network was built on the miRNA expression data of 968 cancer cell lines, which was from CCLE (http://www.broadinstitute.org/ccle). The Pearson correlation coefficient of the miRNA expressions of two cell lines is regarded as the similarity between them, and is used as the weight of the corresponding edge in the network. Protein-protein interactions (PPIs) have been extensively studied, and we used the interactions between the proteins to represent the interactions between the genes coding the proteins. The gene-gene similarity network is based on the PPI data from iRefIndex [31], which contains 2981 genes and 53409 gene-gene interactions, with each gene possessing at least 5 interactions.

The cell line-drug interaction network is based on the drug response data of the 968 cell lines and the 226 drugs from GDSC (http://www.cancerrxgene.org/). The responses have been divided into two types sensitive and resistant according to the log-normalized IC50 threshold, and there are 20346 sensitive responses and 155277 resistant responses. Accordingly, there are 20346 interaction edges with weight 1 in the cell line-drug interaction network.

The copy number variation, somatic mutation and hypermethylation are called Cancer Functional Events (CFEs). The CFE data of the cell lines have been downloaded from GDSC and were used to build the cell line-gene interaction network. Similar to previous literature [32], we classified the cell line-gene relationship into associated and unassociated according to whether the coverage percentage of CFEs in the gene of the cell line is higher than 5%. Finally, we obtained a cell line-gene interaction network of 14330 associations between the 968 cell lines and the 2981 genes. The network is a bipartite graph consisting of gene vertices and cell line vertices and edges with weight 1 indicating corresponding cell line-gene associations.

Finally, we constructed a heterogeneous network including 226 drugs vertices in the drug similarity network, 2981 gene vertices in the gene similarity network, and 968 cell line vertices in the cell line similarity network. The network is represented as a weighted graph $G=(V,E)$. The vertex set of *G* is $V=\{v_1,v_2,\ldots,v_n \}$ whose element denotes a drug, a cell line or a gene. The edge set of *G* is $E=\{e_{i,j} \}$ whose element denotes the relationship between vertex $v_i$ and vertex $v_j$, and the weight of an edge $e_{i,j}$ is set as described above.

### Learning graph representation

As most similar works, we assume that similar cell lines tend to have similar responses to the same drug, and predict the response of a query cell line to a certain drug by utilizing the similarity between the query cell line and other cell lines with known responses to the drug. Since the heterogeneous network has integrated multiple types of information related with cell-lines and drugs, the neighbourhood of cell-lines in the network could be used to measure the similarity between cell-lines. Based on the idea, we use the learning graph representation method GraRep [23] to obtain representation vectors as the topology structure features of the vertices in the network.

Given a graph *G*, Learning Graph Representation (LGR) aims to learn a feature vector $F_i \in R^d$ for vertex $v_i$ such that the global topology structure information (i.e. the neighbourhood) of the vertex is captured in the vector. In our method, the global topology structure information is represented by the distinct connections in different transitional steps between vertices, which is calculated by the process of LGR and is described as follows.

We use an $n \times n$ adjacent matrix *M* to represent the heterogeneous network *G*, and element $M_{ij}$ is the weight of the edge between the vertices $v_i$ and $v_j$. Based on *M*, a weighted degree matrix *W* is calculated according to Eq. (1).

1 $$\begin{aligned} W_{i j}=\left\{ \begin{array}{ll} \sum _{p=1, . ., n} M_{i p}, &{} \text{ if } i=j \\ 0, &{} \text{ if } i \ne j \end{array}\right. \end{aligned}$$

An edge of *G* implies an association relation. Considering transition between vertices, larger $M_{ij}$ means larger transition probability from $v_i$ to $v_j$. Hence the 1-step probability transition matrix *T* is calculated according Eq. (2).

2 $$\begin{aligned} T=W^{-1}M, \end{aligned}$$

where the element $T_{ij}$ is the transition probability from vertex $v_i$ to $v_j$ with exact one step. A *k*-step probability transition matrix $T^k$ is calculated according Eq. (3).

3 $$\begin{aligned} T^{k}=\underbrace{T \cdots T}_{k}, \end{aligned}$$

where $T_{ij}^k$ is the transiting probability from vertex $v_i$ to $v_j$ with exact *k* steps.

For a drug *d* and a cell line *c*, let the representation vectors of *d* and *c* are $\vec {d}$ and $\vec {c}$ respectively, and we model the possibility $P(E=1 \mid d,c)$ that *c* is sensitive to *d* (*i.e.* there is an edge between the vertices *d* and *c* in *G*) as follows:

4 $$\begin{aligned} P(E=1 \mid d, c)=\sigma (\vec {d} \cdot \vec {c})=\frac{1}{1+\textrm{e}^{-\vec{d} \vec{c}}}, \end{aligned}$$

where $\sigma (.)$ is the sigmoid function. Accordingly, $P(E=0 \mid d,c)$ denotes the possibility that *c* is resistant to *d* (*i.e.* there is no edge between the vertices *d* and *c* in *G*) and $P(E=0 \mid d,c)= \sigma ( - \vec {d} \cdot \vec {c} )$.

Our objective is to maximize $P(E=1 \mid d,c)$ for the observed edge (*d*, *c*) in *G* while maximizing $P(E=0 \mid d,c)$ for the resistant response that there is no edge between the vertices *d* and *c*. Therefore, the following log-likelihood $\ell$ of *G* is our global objective function.

5 $$\begin{aligned} \ell =\sum _{d \in V_{D}} \sum _{c \in V_{C}} E(d, c) (\log \sigma (\vec {d} \cdot \vec {c}) + \left. N {\mathbb {E}}_{c_{N} \sim P_{E}}\left[ \log \sigma \left( -\vec {d} \cdot \vec {c}_{N}\right) \right] \right) , \end{aligned}$$

where $V_D$ and $V_C$ are the drug vertex set and the cell line vertex set respectively, and *E*(*d*, *c*) indicates whether there is an edge between the drug vertex *d* and the cell line vertex *c*: $E(d,c) = 1$ if there is an edge, otherwise, it is 0. *N* is the number of resistant responses, and ${\mathbb {E}}$ is the expectation value of the log-likelihood of resistant responses and is defined as Eq. (6).

6 $$\begin{aligned} \begin{array}{l} {\mathbb {E}}_{c_{N} \sim P_{E}}\left[ \log \sigma \left( -\vec {d} \cdot \vec {c}_{N}\right) \right] =\sum _{c_{N} \in V_{C}} p_{E}\left( c_{N}\right) \log \sigma \left( -\vec {d} \cdot \vec {c}_{N}\right) \\ \quad =p_{E}(c) \log \sigma (-\vec {d} \cdot \vec {c})+\sum _{c_{N} \in V_{C} \backslash \{c\}} p_{E}\left( c_{N}\right) \log \sigma \left( -\vec {d} \cdot \vec {c}_{N}\right) , \end{array} \end{aligned}$$

where $p_E (c)$ is the transiting probability from the drug vertex *d* to the cell vertex *c*.

Let $x=\vec {d} \cdot \vec {c}$, and maximizing $\ell$ requires the derivative of $\ell$ with respect to *x* be 0, therefore Eq. (7) follows.

7 $$\begin{aligned} x=\vec {d} \cdot \vec {c}=\log \left( \frac{p_{E}(c \mid d)|E|}{p_{E}(d) p_{E}(c)}\right) -\log N \end{aligned}$$

Let $D_i$ denotes the representation vector of *i*th drug vertex, $C_j$ denotes the representation vector of *j*th cell line vertex, and $Y_{ij}=D_{i} \cdot C_{j}$. According to Eq. (7), we have:

8 $$\begin{aligned} Y_{i j}=D_{i} \cdot C_{j}=\log \left( \frac{T_{i j}}{\sum _{p=1}^{n} T_{i j}}\right) -\log N, \end{aligned}$$

where *T* is the probability transition matrix of graph *G*.

Considering *k*-step random walks and based on Eqs. (3) and (8), we obtain Eq. (9).

9 $$\begin{aligned} Y_{i j}^{k}=\log \left( \frac{T_{i j}^{k}}{\sum _{p=1}^{n} T_{i j}^{k}}\right) -\log N \end{aligned}$$

In order to obtain the representation vectors of the drug vertices and the cell line vertices, we apply a popular singular value decomposition (SVD) method to factorize *Y*.

10 $$\begin{aligned} \left[ U^{k}, \Sigma ^{k},\left( V^{k}\right) ^{T}\right] ={\text {SVD}}\left( Y^{k}\right) \end{aligned}$$

The representation vector matrix of *k*-step random walks $F^k$ is calculated as follows.

11 $$\begin{aligned} F^{k}=U_{d}^{k}\left( \Sigma _{d}^{k}\right) ^{1 / 2} \end{aligned}$$

In Eqs. (10) and (11), $\Sigma _{d}^{k}$ is the matrix composed by the top *d* singular values and $U_d^k$ is the first *d* columns of $U^k$, which are the first *d* eigenvector of $Y^k (Y^k)^T$. For $k = 1$ to *K*, we calculate *K* representation vector matrices, and concatenating them obtains an $n \times K d$ matrix *F*, whose rows are the representation vectors of the vertices in *G*. *F* will be used as the input features in the following classification.

### Classification via support vector machine

As we have assumed before, cell lines with similar topology structures in the network tend to have similar responses to the same drug. Since cell lines with similar topology structures are more similar with respect to the representation vectors, we construct a binary classification model using the representation vectors of the cell lines as the input features and output their responses (sensitive or resistant) to a query drug. To integrate comprehensive similarity information between cell lines, we consider all *k*-step representations with $k =1, 2,\ldots , K$. Using a larger *K* could capture more distant similarity information but also introduce more noise.

In case that drug vertices and genes vertices have few edges with cell line vertices in the heterogeneous network, there would be a large number of poor features for some cell lines. When *K* and *d* are large numbers, the number of features ( i.e., the length $K \times d$ of the representation vector) will be too large, and the overfitting problem will occur. To deal with the problem, we use a Laplacian score method [24] to select out most valuable features from the $K \times d$ features. For each feature, the Laplacian score method assesses the ability to represent the graph structure and calculate a corresponding score. We only use the features with top *t* Laplacian scores for the classification. The values of *K*, *d*, and *t* are determined by 5-fold cross-validation experiments as described earlier in the subsection Parameters Selection.

In the heterogeneous network, the number of sensitive drug responses is 20346, while the number of resistant drug responses is 155277, which is a great imbalance of positive and negative samples. Since the support vector machine (SVM) method could effectively deal with the imbalance problem by assigning different weights to positive and negative samples, we chose SVM to conduct the classification task. In the following experiments, we employed LIBSVM [24] to do the classification. LIBSVM is an integrated software package including diverse SVM models, which can be chosen by setting some options. We set the options of LIBSVM as follows: the SVM_type was set as the default C-SVC, the kernel type was set as polynomial

and the order of the polynomial kernel took the default value 3, the weights for the positive class and the negative class were set as the number of negative samples, and positive samples, respectively, and the other options were left default. For each drug, LIBSVM can learn an SVC model on the training data, and for each cell line, the model outputs a decision score. If the decision score is larger than 0, the cell line is predicted sensitive to the drug, and a larger score indicates that the prediction is more convinced.

## Data Availability

The datasets used and analysed during the current study available from the corresponding author on reasonable request.

## Abbreviations

AUC: Area under receiver operating characteristic curve
AUPRC: Area under precision-recall curve
SVM: Support vector machine
